# Blockade of Wnt/β-catenin signaling suppresses breast cancer metastasis by inhibiting CSC-like phenotype

**DOI:** 10.1038/srep12465

**Published:** 2015-07-23

**Authors:** Gyu-Beom Jang, Ji-Young Kim, Sung-Dae Cho, Ki-Soo Park, Ji-Youn Jung, Hwa-Yong Lee, In-Sun Hong, Jeong-Seok Nam

**Affiliations:** 1 Laboratory of Tumor Suppressor, Lee Gil Ya Cancer and Diabetes Institute, Gachon University, Incheon, 406-840, Republic of Korea; 2 Department of Molecular Medicine, School of Medicine, Gachon University, Incheon 406-840, Republic of Korea; 3 Department of Oral Pathology, School of Dentistry, and Institute of Oral Bioscience, Chonbuk National University, Jeonju, Republic of Korea; 4 Department of New Technology Development, Korea Health Industry Development Institute (KHIDI), Cheongju-si 363-700, Republic of Korea; 5 Department of Companion and Laboratory Animal Science, Kongju National University, Chungnam 314-702, Republic of Korea; 6 The Faculty of Liberal Arts, Jungwon University, Chungbuk, 367-805, Republic of Korea

## Abstract

The identification of cancer stem cells (CSCs) represents an important milestone in the understanding of chemodrug resistance and cancer recurrence. More specifically, some studies have suggested that potential metastasis-initiating cells (MICs) might be present within small CSC populations. The targeting and eradication of these cells represents a potential strategy for significantly improving clinical outcomes. A number of studies have suggested that dysregulation of Wnt/β-catenin signaling occurs in human breast cancer. Consistent with these findings, our previous data have shown that the relative level of Wnt/β-catenin signaling activity in breast cancer stem cells (BCSCs) is significantly higher than that in bulk cancer cells. These results suggest that BCSCs could be sensitive to therapeutic approaches targeting Wnt/β-catenin signaling pathway. In this context, abnormal Wnt/β-catenin signaling activity may be an important clinical feature of breast cancer and a predictor of poor survival. We therefore hypothesized that Wnt/β-catenin signaling might regulate self-renewal and CSC migration, thereby enabling metastasis and systemic tumor dissemination in breast cancer. Here, we investigated the effects of inhibiting Wnt/β-catenin signaling on cancer cell migratory potential by examining the expression of CSC-related genes, and we examined how this pathway links metastatic potential with tumor formation *in vitro* and *in vivo*.

Metastasis/invasion and systemic tumor dissemination from primary tumors are the most detrimental events that occur during cancer progression. It has been hypothesized that a small subpopulation of cancer cells, namely metastasis-initiating cells (MICs), might exist, although these cells have not yet been prospectively identified. Multiple lines of recent evidence strongly suggest that MICs might exist within small subpopulations of cancer stem cells (CSCs) inside of tumors. First, CSCs possess a high tumor-initiating capacity, which is an essential characteristic that enables the formation of new tumors (secondary and tertiary foci) beyond the point at which the original tumor formed^1^,^2^. Second, CSCs express epithelial-mesenchymal transition (EMT) markers^3^, which are associated with the ability of tumor cells to migrate into other tissues or organs. More specifically, some studies have suggested that potential MICs might be present within small CSC populations, for example, CD44^+^/CD24^low^ breast cancer cells with stem cell-like properties have been proposed to exhibit enhanced tumorigenic and metastatic properties in tumor xenograft models^4^,^5^. Interestingly, accumulating evidence indicates a critical role of Wnt/β-catenin signaling in the functioning of CSCs^6^,^7^,^8^. For example, mammary stem cells with high levels of Wnt/β-catenin signaling have a much greater tumorigenic potential than their counterparts with low levels of this type of signaling^9^. Therefore, the findings of these studies suggest that Wnt/β-catenin signaling in MICs may be a promising therapeutic target in breast cancer.

Wnt proteins are a family of secreted, glycosylated, and palmitoylated peptides that mediate a wide variety of processes during embryogenesis by regulating stem cell division, migration, and integrity of the stem cell niche^10^,^11^. Furthermore, aberrant activation of the Wnt/β-catenin signaling pathway has been suggested to play an important regulatory role during the development of several types of human cancers. For example, activating mutations in Wnt/β-catenin signaling components, including β-catenin, Axin, and APC, have been reported in colorectal cancer (CRC) patients^12^ and are also major causes of malignant transformation^13^. Until recently, it was generally assumed that dysregulation of the Wnt/β-catenin signaling pathway rarely, if ever, occurred in breast tumors, in contrast with that in CRC^14^. However, subsequent studies have suggested that in breast cancer, dysregulation of this signaling pathway may be caused by disruption of its negative regulators. Consistent with this notion, expression of the extracellular inhibitor of Wnt/β-catenin signaling, secreted frizzled-related protein 1 (also known as SFRP1), which competes with the Wnt signaling receptor FZD for ligand binding, is significantly down-regulated in many breast cancers and is associated with poor survival and a poor therapeutic response^15^,^16^,^17^. Recently, upregulation of the Wnt/β-catenin signaling pathway has also been shown to lead to increased tumor metastasis from the primary tumor^18^,^19^,^20^.

In the present study, we therefore aimed to explore a limiting factor in metastatic development using a mouse model of breast cancer in which cancer spontaneously metastasizes to the lungs^21^. We hypothesized that Wnt/β-catenin signaling might regulate the self-renewal and migration of CSCs, thereby enabling metastasis and systemic tumor dissemination in breast cancer. Here, we investigated the effects of inhibition of Wnt/β-catenin signaling on cancer cell migration potential by examining the expression of CSC-related genes, and we assessed the manner by which this pathway links metastatic potential with tumor formation. Finally, we showed that inhibition of the Wnt/β-catenin signaling pathway preferentially reduced the metastatic potential by altering CSC activity in a mouse model of breast cancer. Collectively, these data suggest that Wnt/β-catenin signaling is a potential therapeutic target for breast cancer treatment.

## Results

### Wnt/β-catenin signaling activity is enhanced in malignant breast cancer tissues compared with their normal counterparts

**Figure 1:**
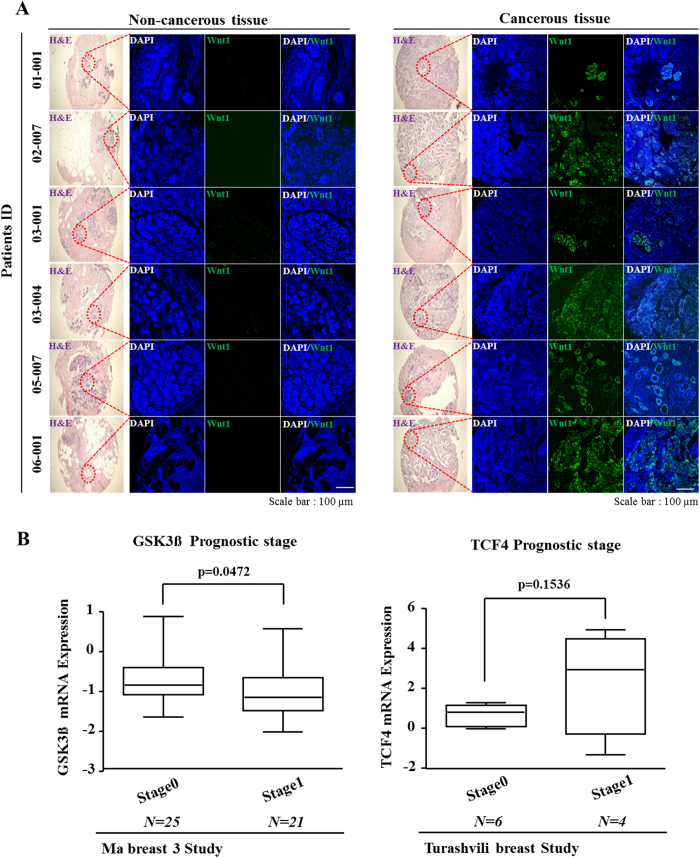
Comparison of Wnt/β-catenin signaling-related genes in malignant breast cancer tissues and their normal counterparts. Cancerous and non-cancerous breast tissues (kindly provided by Dr. Lee at the National Cancer Center, Korea) were stained with antibodies against Wnt1. Wnt1 was expressed to a greater extent in the cancerous tissues than in the non-cancerous tissues. DAPI staining was performed to label the nuclei within each field (**A**). A significant correlation between poor prognosis and the expression of a negative (GSK3β) or positive (TCF4) regulator of Wnt/β-catenin signaling was observed in the human breast cancer datasets assessed, which were obtained through the Oncomine dataset repository (www.oncomine.org) (**B**). The results are presented as the mean ± SD, as determined from more than three independent experiments. *P < 0.05, **P < 0.01, ***P < 0.001.

Recently, accumulating evidence has demonstrated a critical role of Wnt/β-catenin signaling in cancer stemness and malignant behavior^6^,^22^,^23^. Consistent with previous studies, which have shown elevated Wnt/β-catenin signaling activity in different cancer cell models, our immunocytochemical results revealed that Wnt/β-catenin signaling was activated to a greater extent in cancerous tissues compared with that in non-cancerous tissues (Fig. 1A). To further investigate the connection between breast tumorigenesis and Wnt/β-catenin signaling, we evaluated breast cancer datasets available through the Oncomine dataset repository (www.oncomine.org). Filtering specifically for breast cancer datasets that included prognostic stages (categories) based on the size of the tumor and extent of metastasis, we found correlations between cancer stage and the expression of negative (GSK3β) or positive (TCF4) regulators of Wnt/β-catenin signaling (Fig. 1B). These data indicate that Wnt/β-catenin signaling might be associated with poor prognosis and could contribute to the metastatic potential of breast cancer.

### Blockade of Wnt/β-catenin signaling suppresses the growth and phenotypic characteristics of BCSCs

**Figure 2:**
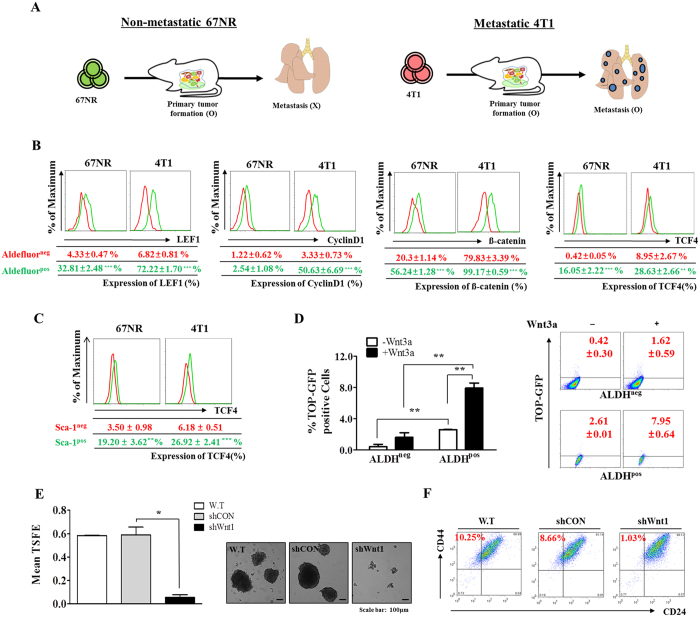
Constitutive activation of the Wnt/β-catenin signaling pathway is a hallmark of tumorigenicity and maintenance of BCSCs. 67NR cells form primary tumors readily, although the tumor cells do not intravasate. On the other hand, 4T1 cells have full metastatic properties (**A**). The percentages of LEF1, cyclin D1, TCF-4, and β-catenin-positive cells in both Aldefluor-positive (**B**) and Sca-1-positive (**C**) subpopulations of non-invasive 67NR cells and highly invasive 4T1 cells were evaluated by flow cytometric analysis (**B,C**). Wnt3a-induced Wnt/β-catenin signaling in ALDH1-positive BCSC subpopulations was assessed using a TOP Flash luciferase reporter. Wnt3a treatment induced transcriptional activity to a greater extent in the ALDH1-positive BCSC subpopulations compared with that in the ALDH1-negative subpopulations (**D**). Wnt1 knockdown inhibited the tumor sphere formation of 4T1 cells. Spheres that were greater than 100 μm in size were enumerated, and a representative image of a tumor sphere is shown. The averages of three independent experiments are shown (**E**). Wnt1 knockdown led to a decrease in the percentage of CD44^+^/CD24^−^ cells as a proportion of the total cancer cells (**F**). Abbreviations: TSFE, tumor sphere-forming efficiency. The results are presented as the mean ± SD, as determined from three independent experiments. *P < 0.05, **P < 0.01, ***P < 0.001.

We used two well-characterized breast cancer cell lines with different metastatic properties to examine the regulatory effects of Wnt/β-catenin signaling on the malignant properties of breast cancer cells^24^, including 67NR cells, which form a primary tumor readily, but tumor cells do not intravasate from the primary tumor, and 4T1 cells, which are fully metastatic and form macroscopic metastatic nodules on the lung surfaces from primary tumors, as illustrated in Fig. 2A. First, to examine the regulatory role of Wnt/β-catenin signaling in BCSCs, we analyzed the expression of the transcription factor TCF-4, a critical regulator of Wnt/β-catenin signaling^25^, in an aldehyde dehydrogenase 1 (ALDH1)-positive subpopulation. Previous studies have demonstrated that ALDH1 activity is a marker of both normal and malignant human mammary stem cells and a predictor of poor clinical outcome^26^,^27^. In this study, we performed Aldefluor flow cytometry-based assay to assess ALDH activity in breast cancer cells. Consistently, the Aldefluor-positive subpopulation showed significantly higher levels of the regulatory components of Wnt/β-catenin signaling, such as LEF1, cyclin D1, β-catenin and TCF-4, compared with those in the Aldefluor-negative subpopulation in both breast cancer cell types (Fig. 2B), suggesting that the BCSC subpopulations exhibited enhanced Wnt/β-catenin signaling activity. Furthermore, recent studies have suggested that the stem cell marker Sca-1^28^ plays an important role in maintaining the pluripotency of BCSCs. To confirm whether Wnt/β-catenin signaling activity is elevated in BCSC subpopulations, we examined TCF-4 expression in an Sca-1-positive subpopulation. Consistent with the above results, the Sca-1-positive subpopulation showed a significantly higher level of TCF-4 compared with the Sca-1-negative subpopulation in the two different breast cancer cell types (Fig. 2C). Moreover, we compared the expression levels of ALDH1 and Sca-1 in non-metastatic 67NR cells with those in highly metastatic 4T1 cells to determine whether Wnt/β-catenin signaling affected the metastatic properties associated with BCSCs. TCF-4 expression was found to be significantly increased in 4T1 cells compared with 67NR cells in both the ALDH1- and Sca-1-positive subpopulations (Fig. 2B,C). These results suggest that Wnt/β-catenin signaling may be important for effective metastasis to distant tissue sites and for tumor growth. Next, to further investigate the correlations between Wnt/β-catenin signaling activity and the ALDH1-positive BCSC subpopulations, 4T1 cells were transiently transfected with a luciferase reporter plasmid in the presence or absence of Wnt3a treatment. Transcriptional activity in the ALDH1-positive BCSC subpopulations was significantly increased by the Wnt3a treatment (Fig. 2D). Recently, it has been suggested that the cancer stem/progenitor cell population may be enriched in three-dimensional (3D) sphere clusters in different types of cancers, including breast^29^, colon^30^, brain, and pancreatic cancers^31^. Therefore, we established a sphere-forming culture system to serve as an *in vitro* BCSC culture model, using our published protocols^32^. To examine whether the blockade of Wnt/β-catenin signaling suppresses tumor sphere formation in breast cancer, we generated stable Wnt1 knockdown 4T1 cells. Short hairpin RNAs (shRNAs) were used to stably suppress Wnt1 expression, and Wnt1 knockdown cells were compared with 4T1 cells expressing non-targeting control shRNAs that were generated at the same time. Successful knockdown of Wnt1 was verified by examining the RNA and protein levels in 4T1 cells (Supplementary Fig. 1A and B). Wnt1 knockdown disrupted the tumor sphere formation of 4T1 cells (Fig. 2E). As expected, under sphere culture conditions, significant shRNA-induced suppression of Wnt1 was clearly observed at the mRNA level (Supplementary Fig. 2). To further confirm the specificity of Wnt1 in tumor sphere formation, we treated cells with Wnt1 ligand with or without Wnt1 knockdown and then evaluated tumor sphere formation. As expected, co-treatment of cells with Wnt1 ligand successfully attenuated the effects of Wnt1 knockdown on tumor sphere formation (Supplementary Fig. 3). In this context, we also examined the expression profiles of BCSC markers in cells with or without Wnt1 knockdown. Specific subpopulations (e.g. CD44^+^/CD24^−^) of breast cancer cells have been reported to have stem/progenitor cell properties^33^,^34^. Consistent with our hypothesis, this BCSC subpopulation was significantly decreased, and Wnt/β-catenin signaling activity was suppressed (Fig. 2F). To further confirm the effects of Wnt/β-catenin signaling on tumor sphere formation and the CD44^+^/CD24^−^ BCSC subpopulation using an alternative method of inhibition, we treated 4T1 cells with another well-known small-molecule Wnt/β-catenin signaling inhibitor, FH535. Approximate IC_50_ values were determined using a dose-response curve. In mouse breast cancer cells, the IC_50_ value was 17 μM (Supplementary Fig. 4). Consistent with the above results (Fig. 2E,F), the FH535 treatment significantly suppressed tumor sphere formation (Supplementary Fig. 5A) and the CD44^+^/CD24^−^ BCSC subpopulation (Supplementary Fig. 5B) in dose-dependent manners.

### Wnt/β-catenin signaling regulates proliferation and apoptosis of breast cancer cells *in vitro*

**Figure 3:**
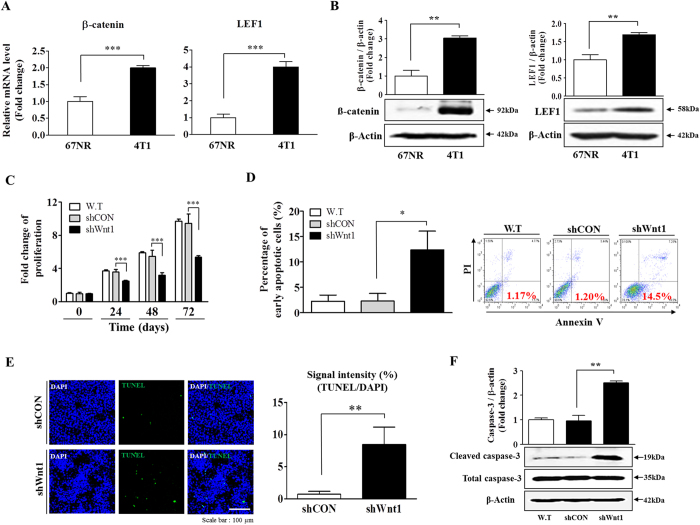
Down-regulation of Wnt/β-catenin signaling suppresses tumor growth. The relative expression of LEF1 and β-catenin, which are downstream components of Wnt/β-catenin signaling, in both non-invasive 67NR cells and highly invasive 4T1 cells was evaluated by real-time PCR and western blotting (**A**,**B**). Transfection of cells with Wnt1 shRNA led to a time-dependent decrease in the number of cells compared with that observed following transfection with control shRNA (**C**). Wnt1 knockdown-mediated cytotoxicity was evaluated by flow cytometry using PE-labeled annexin-V (**D**). Wnt1 knockdown-mediated apoptotic DNA fragmentation and condensation were visualized by TUNEL assay (**E**). The level of activated (cleaved) caspase-3 in cells undergoing Wnt1 knockdown-induced apoptosis was evaluated by western blot, using an antibody targeted against activated caspase-3 (**F**). DAPI staining was performed to label the nuclei within each field. β-actin was used as an internal control. The results are presented as the mean ± SD, as determined from three independent experiments. *P < 0.05, **P < 0.01, ***P < 0.001.

We first compared the expression profiles of downstream signaling components of Wnt/β-catenin signaling, such as β-catenin and LEF1, in non-metastatic 67NR cells versus highly metastatic 4T1 cells to further determine whether this signaling pathway might play a role in this important transition. The protein and mRNA levels of β-catenin and LEF1 were significantly increased in metastatic 4T1 cells compared with non-metastatic 67NR cells (Fig. 3A). To further confirm the significance of Wnt/β-catenin signaling in the metastatic phenotype, we also compared the expression of β-catenin target genes, such as c-Myc and cyclin D1. As expected, the c-Myc and cyclin D mRNA levels were significantly increased in metastatic 4T1 cells compared with non-metastatic 67NR cells (Supplementary Fig. 6A–B). These results suggest that Wnt/β-catenin signaling may be important for effective metastatic growth at distant tissue sites. Furthermore, we compared the expression of other Wnt ligands, such as Wnt3a, Wnt7a, and Wnt10a. Interestingly, within the Wnt family, Wnt3a and Wnt7a were significantly increased in metastatic 4T1 cells compared with non-metastatic 67NR cells (Supplementary Fig. 7A–B), whereas no difference in Wnt10a expression was observed in either cell type (Supplementary Fig. 7C). To assess the effect of Wnt1 knockdown on breast cancer cell growth, cell viability was measured by MTT assay. As shown in Fig. 3C, a time-dependent decrease in the number of Wnt1 knockdown cells was observed compared with that of control shRNA-infected cells. Moreover, to further confirm the specificity of Wnt1 in proliferation, we treated cells with Wnt1 ligand with or without Wnt1 knockdown and than evaluated cancer cell growth. As expected, co-treatment of cells with Wnt1 ligand successfully attenuated the effects of Wnt1 knockdown on proliferation (Supplementary Fig. 8). Flow cytometry assay using PE-labeled annexin-V, TUNEL assay, and western blot analysis were performed to detect activated caspase-3 for evaluation of the effect of Wnt1 knockdown on apoptosis. The apoptotic rate of 4T1 cells transfected with Wnt1 shRNA reached 14.5%, whereas this rate was 1.17% in non-transfected 4T1 cells (Fig. 3D). TUNEL assay and western blot analysis showed similar trends (Fig. 3E,F). To further evaluate the effects of Wnt/β-catenin signaling on breast cancer cell apoptosis using an alternative method of inhibition, we treated 4T1 cells with FH535. Consistent with the above results (Fig. 3D–F), the FH535 treatment caused a significant elevation in the rate of apoptotic cell death in a dose-dependent manner (Supplementary Fig. 5C). Although the molecular mechanism of mediation of breast cancer cell growth by Wnt/β-catenin signaling are still not completely understood, this signaling pathway activates cell growth and cycle regulators, such as cyclin D1^35^,^36^ and Akt^37^,^38^, which are known to play essential roles in proliferation in various types of cancer. Therefore, to identify potential downstream regulators of Wnt/β-catenin signaling, we analyzed the expression of cell growth and cycle regulators, such as Akt and cyclin D1, with or without Wnt1 knockdown. Interestingly, cyclin D1 expression (Supplementary Fig. 9A) and Akt phosphorylation (Supplementary Fig. 9B) were significantly lower in the Wnt1 knockdown groups, suggesting that Wnt/β-catenin signaling may positively regulate breast cancer growth through activation of the cyclin D1 and Akt signaling pathways (Supplementary Fig. 9C).

### Blockade of Wnt/β-catenin signaling suppresses breast cancer invasion and migration

**Figure 4:**
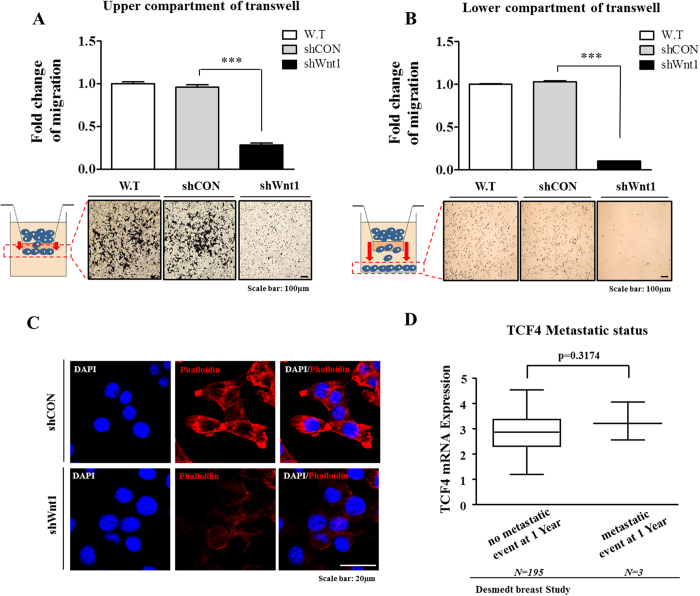
The Wnt/β-catenin signaling pathway regulates tumor cell invasion. Cell migration ability was evaluated by transwell migration assay. Transfection with Wnt1 shRNA significantly decreased 4T1 cell migration across the membrane in both the upper and lower compartments of transwells compared with that observed following transfection with control shRNA (**A**,**B**). Wnt1 knockdown-induced fiber disorganization and full morphological transition were visualized by actin-phalloidin staining (**C**). A significant correlation between metastatic malignancy and the expression of a positive (TCF4) regulator of Wnt/β-catenin signaling was observed in human breast cancer datasets that were obtained through the Oncomine dataset repository (www.oncomine.org) (**D**). DAPI staining was performed to label the nuclei within each field. The results are presented as the mean ± SD, as determined from three independent experiments. *P < 0.05, **P < 0.01, ***P < 0.001.

We investigated the roles of Wnt1 in the invasion and migration of breast cancer cells by transwell migration assay. Cells that migrated across the membrane were stained purple and counted. The results showed that following Wnt1 knockdown, the ability of cells to migrate across the transwell membrane was significantly decreased in both the upper and lower compartments of the transwell unit (Fig. 4A,B), suggesting that Wnt/β-catenin signaling is necessary for migration and therefore might play a role in breast cancer metastasis. Consistent with the results of the Wnt1 knockdown experiment, FH535 treatment significantly suppressed the migratory ability of cells across the transwell membrane (Supplementary Fig. 4D). Moreover, to further confirm the specificity of Wnt1 in breast cancer metastasis, we treated cells with Wnt1 ligand with or without Wnt1 knockdown and then evaluated cancer cell metastasis. As expected, co-treatment of cells with Wnt1 ligand successfully attenuated the effects of Wnt1 knockdown on metastasis (Supplementary Fig. 10). Previous studies have indicated that the actin cytoskeleton is required for tumor cell migration because it pushes or pulls on substrates near cell membranes^39^. Therefore, we examined the distribution of the actin cytoskeleton at the subcellular level in 4T1 cells following Wnt1 knockdown. Actin-phalloidin staining revealed a strong correlation between Wnt1 knockdown and a highly disorganized actin cytoskeleton (Fig. 4C), suggesting that the reduced migration of Wnt1 knockdown cells may be related to disorganization of the actin cytoskeleton. Distinct patterns of actin organization are also regulated by the small GTP-binding protein Rac^40^. Therefore, we additionally performed immunofluorescence staining to analyze Rac expression. Consistent with the disappearance of phalloidin, the amount of the activated form of Rac was decreased in Wnt1 knockdown cells (Supplementary Fig. 11). To further investigate the connection between migration and Wnt/β-catenin signaling, we evaluated breast cancer datasets available through the Oncomine dataset repository (www.oncomine.org). Filtering specifically for breast cancer datasets including the risk of metastasis during the first year following treatment, we found correlations between metastatic malignancy and the expression of positive regulators of Wnt/β-catenin signaling (e.g., TCF4) (Fig. 4D). These data indicate that Wnt/β-catenin signaling might be associated with the metastatic potential of breast cancer.

### Blockade of Wnt/β-catenin signaling leads to reduced tumor growth in a murine xenograft model

**Figure 5:**
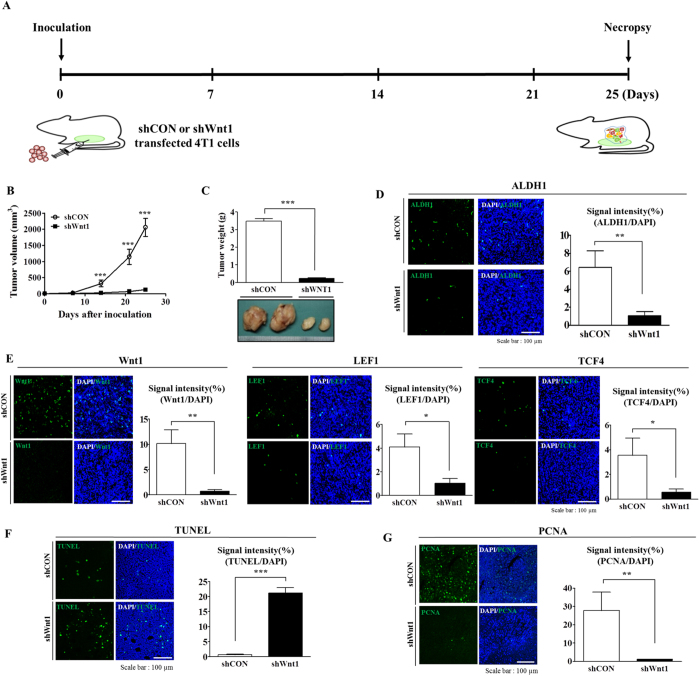
A blockade of Wnt/β-catenin signaling suppresses tumorigenesis in a murine xenograft model. A schematic representation of the experimental protocol, as described in the Materials and Methods section (**A**). Mice were implanted with 4T1 cells (5 × 10^4^ cells/mouse) by orthotopic injection into the thoracic mammary fat pads. Tumor tissues were isolated from mice bearing 4T1 or MDA-MD-435 cells transfected with Wnt1 shRNA or control shRNA. Tumor volumes were measured, as described in the Materials and Methods section (**B**,**C**). The ALDH-positive subpopulation, as a proportion of the total cell population in the tumor xenografts, was assessed by immunohistochemistry (**D**). The relative expression of downstream components of Wnt/β-catenin signaling, such as Wnt1, LEF1, and β-catenin, in bulk tumors was assessed by immunohistochemistry (**E**). Wnt1 knockdown-mediated apoptotic DNA fragmentation in tumor xenografts was visualized by TUNEL assay (**F**). Tumorigenesis promoted by Wnt/β-catenin signaling was further confirmed by performing proliferating cell nuclear antigen (PCNA) immunohistochemistry to assess tumor xenografts (**G**). DAPI staining was carried out to label the nuclei within each field. The results are presented as the mean ± SD, as determined from three independent experiments. *P < 0.05, **P < 0.01, ***P < 0.001.

Following our *in vitro* experiments, we further investigated the *in vivo* efficacy of Wnt1 knockdown on tumorigenesis using a mouse xenograft model. Wnt1 knockdown 4T1 cells were injected into the mammary fat pads of female BALB/c mice, and tumor formation was monitored. Importantly, there was a consistent and significant reduction in tumor outgrowth in the mice injected with Wnt1 knockdown cells compared with those injected with control cells (Fig. 5A–C). Previous studies have demonstrated that ALDH1 is a marker of both normal and malignant human mammary stem cells and a predictor of clinical outcome^26^,^27^. Consistent with the above results, the ALDH1-positive subpopulation showed a significantly higher level of TCF-4 (a positive regulator of Wnt/β-catenin signaling) compared with that in the ALDH1-negative subpopulation in two different breast cancer cell types (Fig. 2B), suggesting that the BCSC subpopulations exhibited enhanced Wnt/β-catenin signaling activity. Therefore, to determine whether and to what extent Wnt1 knockdown affects the proportion of BCSCs *in vivo*, we examined the expression profiles of Wnt/β-catenin signaling components, such as Wnt1, LEF1, and TCF4, in tumors with or without Wnt1 knockdown. Indeed, Wnt1 knockdown led to a decrease in the ALDH1-positive (Fig. 5D) and Wnt/β-catenin signaling component-positive subpopulations (Fig. 5E). Consistent with these results, the expression levels of β-catenin target genes, including Axin2 and cyclin D1, were markedly lower in the Wnt1 knockdown group compared with those in the non-transfected group (Supplementary Fig. 12A–B). Moreover, Wnt1 knockdown induced the cytoplasmic accumulation of β-catenin in breast cancer cells (Supplementary Fig. 12C). Additionally, to further confirm whether Wnt1-positive breast tumor cells represent an LEF1-positive subpopulation, we investigated the co-expression of these markers in tumor xenografts. As shown in Supplementary Fig. 13, we confirmed that Wnt1-positive populations mostly overlapped with LEF1-positive subpopulations in tumor xenografts. Promotion of tumourigenesis by Wnt/β-catenin signaling was further confirmed by TUNEL assay (Fig. 5F) and proliferating cell nuclear antigen (PCNA) immunohistochemistry (Fig. 5G) using tumor xenografts.

### Blockade of Wnt/β-catenin signaling reduces tumor metastasis in a murine xenograft model

**Figure 6:**
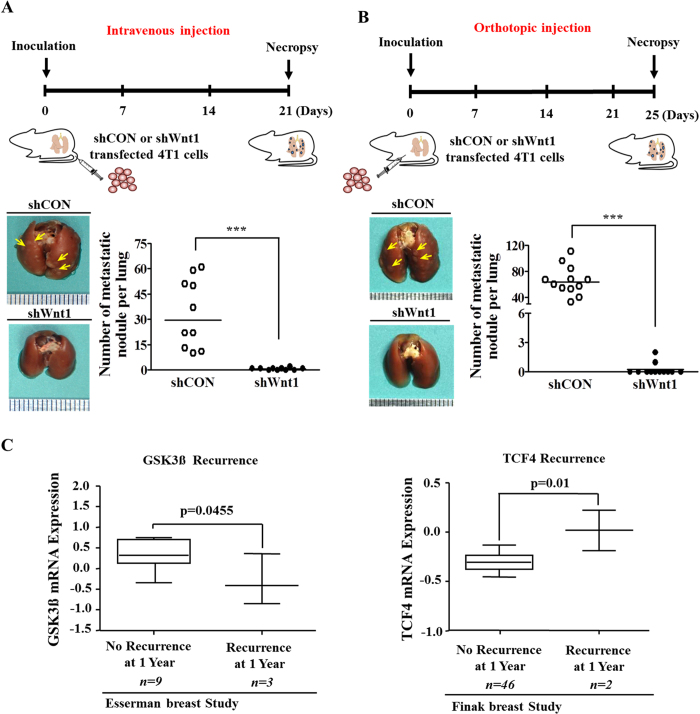
The Wnt/β-catenin signaling pathway regulates tumor metastasis in a murine xenograft model. Mice were implanted with 4T1 cells (5 × 10^4^ cells/mouse) by intravenous injection (**A**) or orthotopic injection into the mammary fat pads (**B**). Metastatic colonization of the lungs was measured as described in the Materials and Methods section (n = 10). A significant correlation between poor prognosis and the expression of a negative (GSK3β) or positive (TCF4) regulator of Wnt/β-catenin signaling was observed in human breast cancer datasets obtained through the Oncomine dataset repository (www.oncomine.org) (**C**). A significant correlation between the risk of recurrence and the expression of a negative (GSK3β) or positive (TCF4) regulator of Wnt/β-catenin signaling was observed in human breast cancer datasets obtained through the Oncomine dataset repository (www.oncomine.org) (**C**). The results are presented as the mean ± SD, as determined from more than three independent experiments. *P < 0.05, **P < 0.01, ***P < 0.001.

An ideal and truly curative breast cancer treatment would prevent distant metastasis, thereby preventing disease relapse. Breast cancer can spread from its primary site (the breast) to other sites throughout the body, but it primarily metastasizes to the bone, lungs, liver, brain, and lymph nodes, and the lungs are among the most common sites of metastasis^41^. Therefore, to further confirm the connection between Wnt/β-catenin signaling and breast cancer metastasis observed *in vitro*, we assessed the effects of Wnt1 knockdown on metastasis using *in vivo* metastatic models of 4T1 cells. Cell lines expressing control non-targeting shRNA and Wnt1 shRNA were injected intravenously (Fig. 6A) or orthotopically into the mammary fat pads (Fig. 6B) of female BALB/c mice, which were monitored for tumor metastasis. Metastatic colonization of the lungs was significantly reduced in the Wnt1 shRNA-transfected groups compared with the control shRNA-transfected groups in both metastatic tumor models. To investigate the connection between metastasis and Wnt/β-catenin signaling, we evaluated breast cancer datasets available through the Oncomine dataset repository (www.oncomine.org). Filtering specifically for breast cancer datasets that included the recurrence risk during the first year following treatment, we found significant correlations between recurrence and the expression of negative (GSK3β) or positive (TCF4) regulators of Wnt/β-catenin signaling (Fig. 6C). These results indicated that the blockade of Wnt/β-catenin signaling may suppress the ability of breast cancer cells to metastasize (travel) to distant tissues or organs.

## Discussion

Approximately 30% to 50% of patients recently diagnosed with early stage breast cancer are likely to progress to the metastatic stage despite receiving treatment, such as surgery and/or chemotherapy^42^. In this context, the identification of CSCs represents an important milestone in the understanding of chemodrug resistance and cancer recurrence^43^. Considering the characteristics of CSCs, the targeting and eradication of these cells represents a potential strategy for significantly improving clinical outcomes. Recent advances in the understanding of the biological characteristics of BCSCs have facilitated the identification of mechanisms underlying the development of malignant breast cancer. A number of studies have suggested that dysregulation of Wnt/β-catenin signaling occurs in human breast cancer^44^. Consistent with these findings, our previous data have shown that the relative level of Wnt/β-catenin signaling is significantly higher in BCSCs compared with that in bulk cancer cells. These results suggest that BCSCs could be sensitive to therapeutic approaches that target Wnt/β-catenin signaling pathway. In this context, abnormal Wnt/β-catenin signaling activity may be an important clinical and pathologic feature of breast cancer and a predictor of poor overall survival^45^.

This study showed that suppression of Wnt/β-catenin signaling by shRNA-mediated Wnt1 silencing in 4T1 cells resulted in 1) decreased levels of the stem cell markers ALDH1, Sca-1, and CD44^+^/CD24^−^; 2) reduced BCSC sphere formation; 3) suppressed growth *in vitro* and *in vivo*; and 4) reduced migration *in vitro* and *in vivo*. These results indicate that Wnt/β-catenin signaling may play a critical regulatory role in the promotion of lung tumorigenesis by stimulating tumor growth, altering the phenotypes of BCSCs, and promoting migration and invasion of breast cancer cells. In this study, we used a combination of several molecular biological approaches to determine the function of the Wnt/β-catenin signaling pathway in BCSCs. Flow cytometric analysis revealed that shRNA-mediated Wnt1 silencing in 4T1 cells resulted in reduced BCSC sphere formation as well as decreases in ALDH1- and Sca1-positive cell subpopulations. Consistent with our results, recent studies have also suggested that the stem cell markers Sca-1^28^ and ALDH1^46^ are responsible for maintaining the pluripotency of BCSCs and for initiating tumor formation *in vivo*. In addition, activity of the functional marker ALDH is highly correlated with an aggressive BCSC phenotype and poor overall survival in patients with primary breast carcinoma^47^. Furthermore, we found that Wnt1 knockdown significantly reduced the CD44^+^/CD24^−^ subpopulation in 4T1 cells (Fig. 2F). These findings suggest that Wnt/β-catenin signaling is a critical regulator of BCSC self-renewal and pluripotency, and they are in accordance with the observed upregulation of Wnt/β-catenin signaling in malignant breast cancer tissues (Fig. 1A).

CSCs, which are responsible for resistance to chemotherapy and cancer recurrence after radiation, are thought to be enriched under low-adherent sphere-forming conditions^48^,^49^. It is well known that cancer cells with CD44^+^/CD24^low^ expression within breast tumors exhibit malignant behaviors^4^,^33^. In a recent study performed using *in vivo* and *in vitro* experimental model systems, a high percentage of BCSCs with the CD44^+^/CD24^low^ phenotype was observed in a model with an increased ability to metastasize via the lymphatic route^50^. Notably, the CD44^+^/CD24^−^ subpopulation in breast cancer cells is enriched under suspension sphere culture conditions^51^. As shown in Fig. 2F, the CD44^+^/CD24^−^ fraction in control-shRNA transfected cells (8.66%) was significantly reduced by Wnt1 knockdown in this study (1.03%). Therefore, reductions in the CD44^+^/CD24^−^ subpopulation appear to be correlated with reduced BCSC sphere formation in Wnt1-depleted 4T1 cells.

In this study, we also functionally established a connection between BCSC-related Wnt/β-catenin signaling and apoptosis/migration. Importantly, the reduced expression of various stem cell markers, such as CD44^+^/CD24^−^, ALDH1, and Sca-1, in Wnt1-depleted cells was correlated with a functional loss of the stem cell characteristics of BCSCs, and shRNA-mediated Wnt1 knockdown in 4T1 cells significantly reduced their growth potential (Fig. 3C) and resistance to apoptosis (Fig. 3D–F). Moreover, remodeling of the actin cytoskeleton is required for tumor cell migration because the cytoskeleton pushes or pulls on substrates near cell membranes, and such changes are induced by Wnt1 knockdown. We stained for actin-phalloidin and found that filopodial activity was severely reduced in these cells (Fig. 4C). In agreement with this finding, we also showed that Wnt1 depletion dramatically down-regulated migration rates toward the bottom of the transwell (Fig. 4A,B). These changes resulted in accelerated migration and may have led to increased metastasis *in vivo* (Fig. 6A,B).

**Figure 7:**
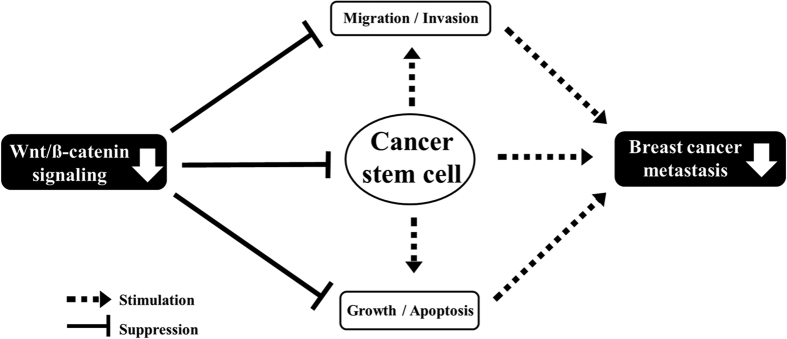
Schematic summary of the role of the Wnt/β-catenin signaling pathway in the development of metastatic breast cancer. Wnt/β-catenin signaling regulates the self-renewal and migration of CSCs, thereby promoting tumor growth and metastasis/systemic dissemination in breast cancer.

Therefore, to further confirm the connection between Wnt/β-catenin signaling and breast cancer metastasis *in vitro*, we assessed the effects of Wnt1 knockdown on metastasis using *in vivo* metastatic models of 4T1 cells. Currently, the *in vivo* tumor models that are most commonly used to study the process of cancer metastasis to the lung primarily rely on the introduction of tumor cells directly into systemic circulation by injection into the tail veins of laboratory rodents^52^,^53^,^54^. Although intravenous administration is useful for identifying factors associated with the growth of cancer cells that have been recruited to the lungs from systemic blood circulation, it does not encompass all the events that are essential for the dissemination process from the primary tumor. Considering these disadvantages, we employed an additional mouse model of breast cancer metastasis that more accurately reflected the metastatic process of this type of cancer. In this model, cancer cells were orthotopically implanted into the mammary fat pads of mice. We found that metastatic colonization of the lungs was significantly reduced in the Wnt1 shRNA-transfected groups compared with that in the control shRNA-infected groups in both metastatic tumor models (Fig. 6A,B). These findings support the importance of Wnt/β-catenin signaling in breast cancer metastasis *in vitro*. Such mechanisms function to prevent breast cancer metastasis because migration is thought to be essential to the metastatic process. Transformed breast cancer cells must possess the ability to leave the initial tumor site, migrate and penetrate into neighboring tissues. In conclusion, we have demonstrated that Wnt/β-catenin signaling regulates the self-renewal and migration of CSCs, thereby promoting tumor growth and metastasis/systemic dissemination in breast cancer, as illustrated in Fig. 7. Taken together, our data suggest that Wnt/β-catenin signaling could serve as a novel target in BCSCs for the treatment of breast cancer.

## Methods

### Cell culture and reagents

Murine mammary cancer cell line 4T1 and 67NR^55^ were cultured in DMEM (Invitrogen, Grand Island, NY) supplemented with 10% fetal bovine serum (FBS), 100 U/ml penicillin and 100 U/ml streptomycin (Lonza, Basel, Switzerland) at 37 °C and 5% CO_2_.

### Short hairpin RNA

Small hairpin RNA (shRNA) targeting mouse Wnt1 and non-targeting RNA were purchased from Sigma (St. Louis, MO, USA). For the efficient Wnt1 shRNA transfection, reverse transfection was performed using Lipofectamine 2000 (Invitrogen) according to the manufacturer’s instructions. Briefly, 293FT cells were transfected with the transfer vector plasmid pLKO.1-shWnt1. The supernatants were harvested 48h after transfection, pooled, and the lentiviral stocks were stored in small aliquots at −80 °C for titration and cell infection. Lentiviruses were diluted in 2 ml DMEM containing polybrene (6 μg/ml). Stable transfectants were selected by incubation with puromycin (2 lg/ml; Sigma–Aldrich). We chose the Wnt1 shRNA that is most effective in mRNA levels from five shRNA designed from the target sequence and determined by qRT-PCR.

### Tumorsphere formation

Single cells were resuspended in serum-free DMEM (Invitrogen) containing B27 (Invitrogen), 20 ng/ml EGF, 20 ng/ml bFGF (PeproTech) and 4 μg/ml heparin (Sigma-Aldrich). Primary tumorspheres were derived by plating 50,000 single cells/well into six-well ultra-low attachment dishes (Corning). Individual spheres ≥ 100 μm from each replicate well (n ≥ 9 wells) were counted under an inverted microscope at 50X magnification using the Image-Pro Plus program (Media Cybernetics). The percentage of cells capable of forming spheres, termed the ‘tumorsphere formation efficiency (TSFE)’, was calculated as follows: [(number of sphere formed/number of single cells plated) X 100].

### Cell proliferation (Cytotoxicity) assay

4T1 cells were seeded in 96-well plates. After 48 h of incubation, cell viability was assessed by cell counting kit-8 (Dojindo) according to the manufacturer’s instruction. The numbers of viable cells were measured at a wavelength of 450 nm using Versamax microplate reader.

### Real-time PCR

Total RNA was extracted from the cells using TRIzol reagent (Invitrogen, USA) according to the manufacturer’s instructions. Complementary DNAs (cDNAs) by adding purified RNA and oligo-dT primers to SuperScript II (Invitrogen). Real-time quantitative PCR was performed using SYBR Green (Applied Biosystems, USA) according to the manufacturer’s protocol. Real-time PCR was performed using a Rotor-Gene Q (Qiagen). Relative mRNA expression of selected genes was normalized to HPRT. The sequences of the primer sets used for this study are provided in Supple. Table 1.

### Flow cytometry

FACS analysis and cell sorting were performed using FACS Calibur and FACS Aria machines (Becton Dickinson, Palo Alto, CA), respectively. FACS data were analyzed using Flowjo software (Tree Star, Ashland, OR). Antibodies to the following proteins were used: PE-conjugated Sca-1 (dilution 1/20), CD44 (dilution 1/40), and CD24 (dilution 1/40). The FACS gates were established by staining with isotype antibody or secondary antibody. The Aldefluor kit (Stem Cell Technologies) was used to isolate the population with a high ALDH enzymatic activity. Cells were stained for ALDH using the Aldefluor reagent according to the manufacturer’s instructions and analyzed on FACS Calibur. As negative control, for each sample of cell aliquot was treated with 50 μM DEAB, a specific ALDH inhibitor. Aldefluor^pos^ cells were quantified by calculating the percentage of total fluorescent cells compared with a control staining reaction. FACS Aria was used to sort Aldefluor-stained cells into Aldefluor^neg^ and Aldefluor^pos^ cell population.

### Luciferase reporter assay

Cancer cells were seeded at a density of 2 × 10^4^ cells/well in 48-well plates. Luciferase reporter assay based on TOP/FOPflash reporter plasmid system was used for the detection of Wnt/ß-catenin transcriptional activity. Luciferase reporter gene analysis was performed in control and Wnt3a-treated (100 ng/ml) 4T1 cells. Luciferase has a secretory signal that is secreted into the cell medium. Luciferase activity was measured by a luminometer (Promega), using a dual-Luciferase assay kit (Promega), according to the manufacturer’s recommendations. Each assay was performed in triplicate and the reporter activity was expressed as mean ± SD.

### Western blot analysis

After the indicated treatments, cells were lysed in ice-cold RIPA buffer containing protease inhibitor cocktail (Sigma Chemical, St. Louis, MO). The concentration of protein was measured by Bio-Rad protein assay kit according to the manufacturer’s protocol. The protein samples separated by SDS-PAGE were then transferred onto nitrocellulose membranes. The membranes were blocked in PBS containing 5% skim milk, and then incubated overnight at 4 °C with the specific primary antibodies of interest for protein detection. Secondary antibodies were then added to each membrane, incubated for 2 h at 37 °C. The presence of target proteins was detected using ECL system. Each band on western blotting was quantified with β-actin as the internal control.

### Immunofluorescent staining

The use of fresh breast tumor specimens was approved by the research ethic committees at the Korea National Cancer Center (NCC). Informed consent was obtained from all patients. All experiments with human specimens were performed in accordance with relevant guidelines and regulations of NCC. Samples were fixed with 4% paraformaldehyde for fluorescent staining. Samples were permeabilized with 0.3 M glycine and 0.3% Triton X-100, and nonspecific binding was blocked with 2% normal swine serum (DAKO, Glostrup, Denmark). Staining was performed as described previously^56^, using the primary anti-Wnt1 (Abcam), anti-Phalloidin (Cytoskeleton Inc.), anti-ALDH1 (Abcam), anti-TCF41 (Abcam), anti-PCNA (Abcam), and anti-LEF1 (Cell Signaling Technology). Samples were examined by fluorescence microscopy (Zeiss LSM 510 Meta). The calculation of expression was based on green fluorescence area and density divided by cell number, as determined from the number of DAPI-stained nuclei, in three randomly selected fields for each specimen from a total of three independent experiments. For quantitation, an arbitrary threshold was set to distinguish specific from background staining, and this same threshold setting was applied to all the samples analyzed.

### TUNEL assay

DNA strand breaks in apoptotic cells were measured with a TUNEL assay using the *In-situ* Detection Kit (Roche Molecular Biochemicals). The samples were fixed with 4% paraformaldehyde in PBS for 15 min and incubated in a 0.1% ice-cold Triton X-100 solution for permeabilization for 10 min according to the manufacturer’s instructions. The cells were then washed 3 times with PBS and incubated with 50 μl of TUNEL reaction mixture at 37 °C for 60 min in a dark, humidified chamber. The cells were then rinsed three times in PBS. The results were visualized by fluorescent microscopy.

### *In vitro* cell migration assay

Cell were plated at 1 × 10^5^ cells/well in 200 μL of culture medium in the upper chamber of Transwell permeable supports (Corning Inc, Corning, NY) with 8.0-μm pore, polycarbonate membrane, 6.5-mm diameter, and 24-well plate format) to track migration of 4T1 cells. The cells on the upper surface of the membranes were completely removed by using a cotton swab. Migrated cells on the lower surface of the membranes were fixed with 4% paraformaldehyde for 10 min, stained with hematoxylin (Sigma-Aldrich), and later the number of cells was counted in three randomly selected fields of the wells under light microscope. To calculate the chemotactic index, the number of cells migrated in response to Wnt1 knockdown was divided by the number of spontaneously migrated cells (control).

### Tumorigenesis experiment

All mice were maintained according to Institutional Animal Care and Use Committee (IACUC)-approved protocols of the Lee Gil Ya Cancer and Diabetes Institute (No.LCDI-2012-0069). For tumorigenesis experiments, anesthetized 7-week-old female Balb/c (Orient Charles River Technology, Korea) were inoculated with 5 × 10^4^ 4T1 cells into the mammary fat pads in 50 μL volume (n = 10 for each group). After inoculation, the mice were randomly assigned to knockdown groups and control group. And it was monitored for 4 weeks. Weight of tumors (n = 10) were determined independently by two observers to assess inter-observer variation. The tumor volume was measured along the two diameter axis with calibers to allow a calculation of the tumor volume, V = (LxW^2^)/2, where L and W are the larger and smaller diameters, respectively.

### Lung metastasis animal model

For the metastasis experiment, 9-week-old female BALB/c mice were inoculated with 5 × 10^4^ 4T1 cells intravenously or orthotopically into the mammary fat pads of female BALB/c mice. After inoculation, the mice were randomly assigned to knockdown groups and control group. Mice were euthanized and lungs were collected on 4 weeks. Macroscopic quantitation of metastases was performed by counting the number of tumor nodules on the lung surface.

### Statistical analysis

All the statistical data were analyzed by GraphPad Prism 5.0 (GraphPad Software, San Diego, CA) and evaluated by two-tailed Student’s t-test. Value of P < 0.05 was considered to indicate statistical significance.

## Additional Information

**How to cite this article**: Jang, G.-B. *et al.* Blockade of Wnt/β-catenin signaling suppresses breast cancer metastasis by inhibiting CSC-like phenotype. *Sci. Rep.* **5**, 12465; doi: 10.1038/srep12465 (2015).

## Supplementary Material

Supplementary Information
