# Supplementary material for: Blockade of Wnt/β-catenin signaling suppresses breast cancer metastasis by inhibiting CSC-like phenotype
Source: Sci Rep. 2015 Jul 23;5:12465. doi: 10.1038/srep12465 (PMC5378883; doi:10.1038/srep12465)
Supplement: Supplementary Information [file srep12465-s1.pdf]

Supplementary information

Title: Blockade of Wnt/β-catenin signaling suppresses breast cancer metastasis by inhibiting CSC-like phenotypes

Authors: Gyu-Beom Jang, Ji-Young Kim, Sung-Dae Cho, Ki-Soo Park, Ji-Youn Jung, Hwa-Yong Lee, In-Sun Hong, Jeong-Seok Nam

Supplement Figure. 1

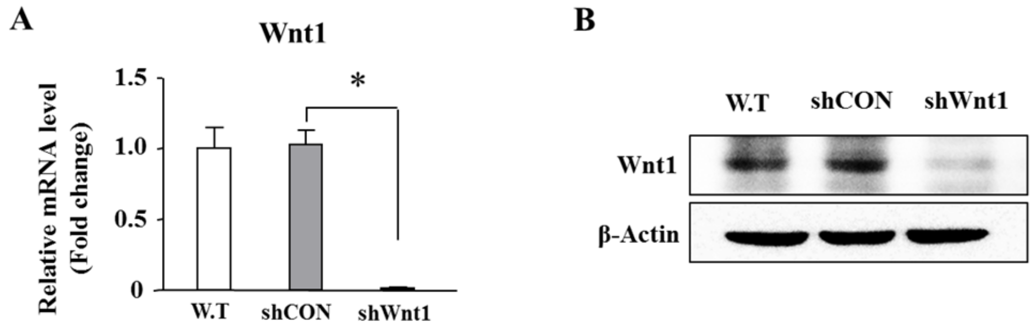

Supplemental Figure 1. Knockdown efficiency of shRNAs targeting Wnt1. 4T1 cells were stably transduced with shRNA targeting Wnt1 or with a non-targeting control shRNA. Successful knockdown of Wnt1 was verified based on RNA **(A)** and protein levels **(B)** in 4T1 cells. β-actin was used as the internal control. The results are the mean ± SD from three independent experiments. * $P<0.05$, ** $P<0.01$, *** $P<0.001$.

Supplement Figure. 2

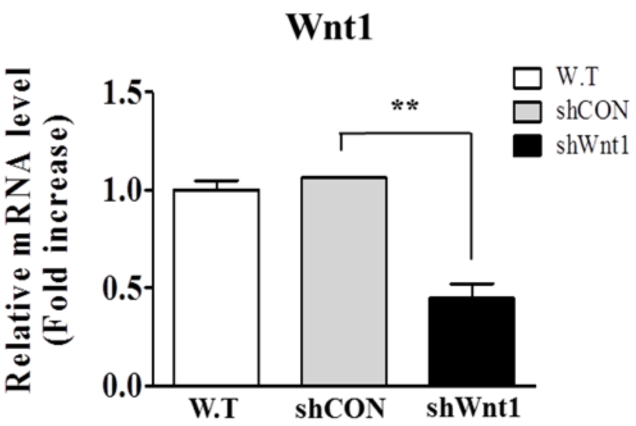

Supplementary figure 2. The verification of Wnt1knockdown at mRNA level under sphere culture condition. 4T1 cells were stably transduced with shRNA targeting Wnt1 or with a non-targeting control shRNA. Successful knockdown of Wnt1 was verified based on RNA levels in 4T1 cells. The results are the mean ± SD from three independent experiments. * P<0.05, ** P<0.01, *** P<0.001.

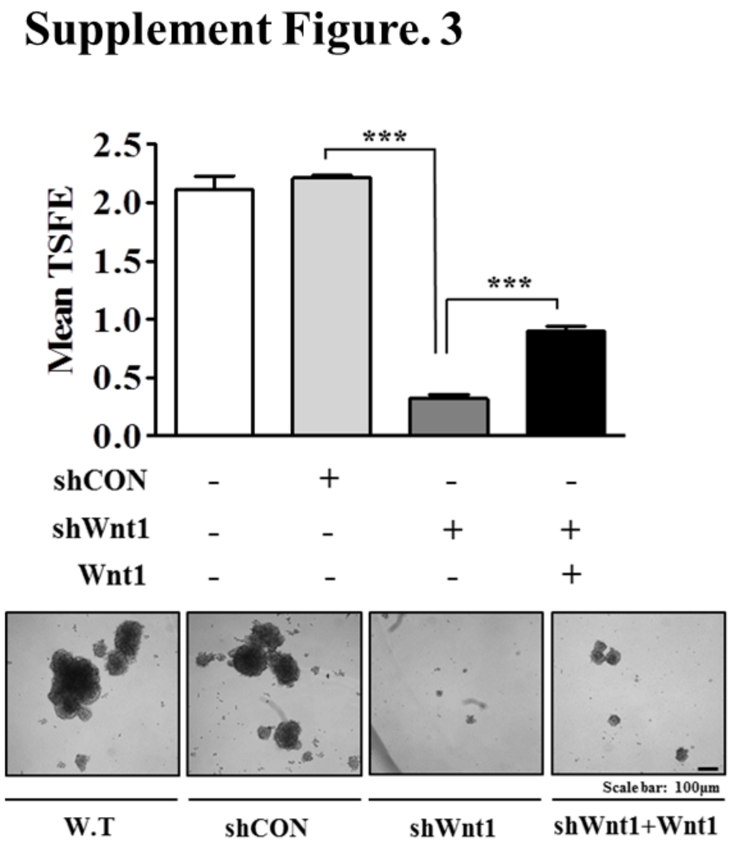

Supplementary figure 3. The specificity of Wnt1 in tumor sphere formation. Co-treatment of cells with Wnt1 ligand successfully attenuated Wnt1 knockdown-mediated effects on the tumor sphere formation. The spheres greater than 100 μm in size were enumerated; a representative image of a tumor sphere is shown. The averages of three independent experiments are shown. Abbreviations: TSFE, tumor sphere-forming efficiency. The results are the mean ± SD from three independent experiments. * $P<0.05$, ** $P<0.01$, *** $P<0.001$.

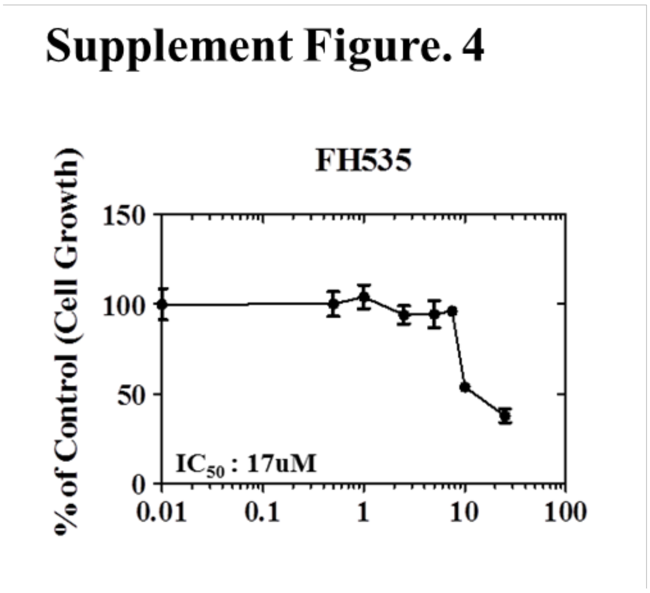

Supplementary figure 4. The treatment concentration that inhibits 50% of the cell proliferation (IC50). The inhibition of cell viability through FH535 treatment for 48 hours was determined using a CCK-8 assay (mitochondrial dehydrogenase activity) in 4T1 cells. The cell viability (%) was calculated as a percent of the vehicle control.

Supplement Figure. 5

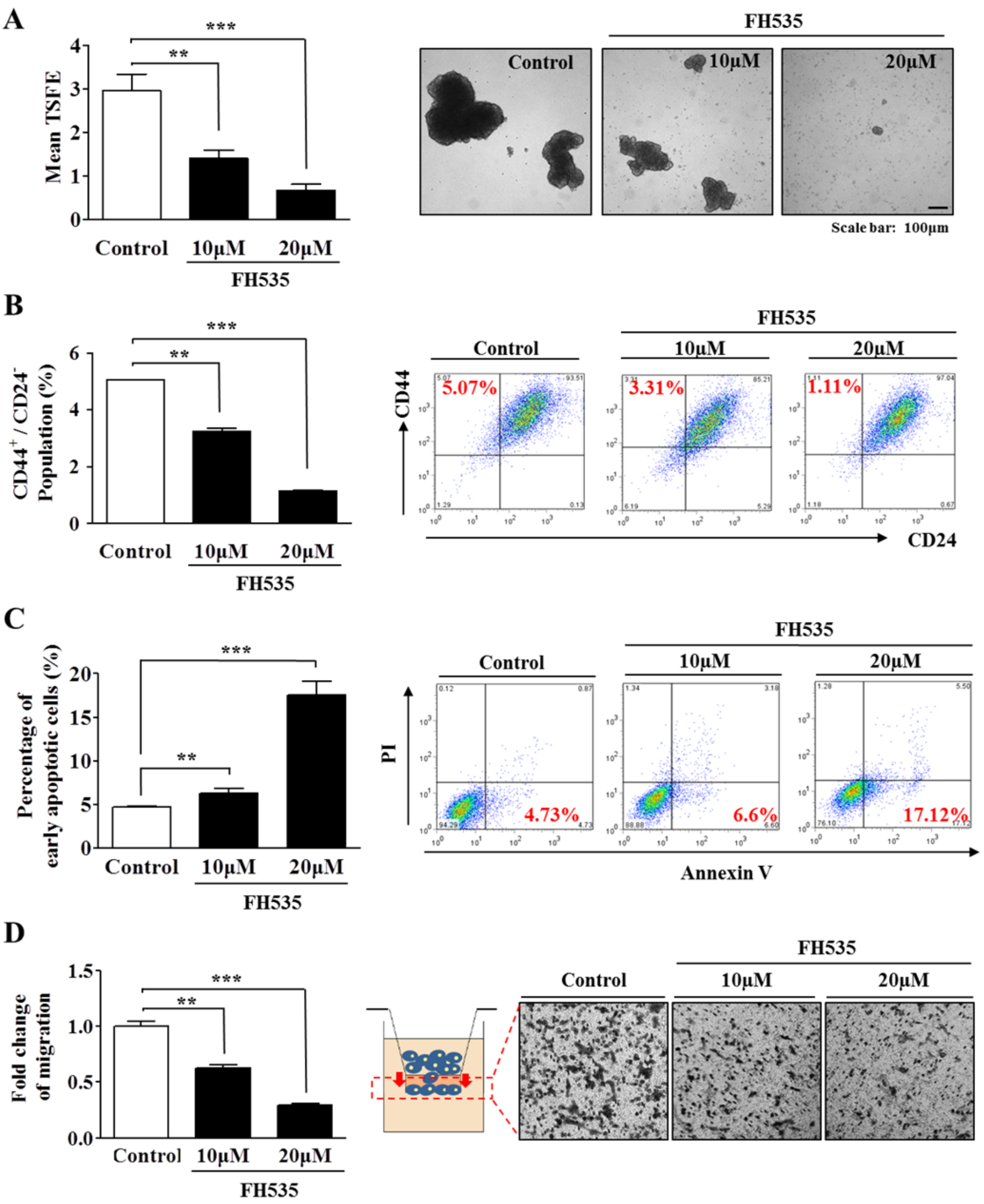

Supplementary figure 5. The effects of Wnt/β-catenin signaling inhibitor FH535 on sphere formation, $CD44^{+}/CD24^{-}$ BCSC subpopulation, apoptosis, and migration. FH535 treatment inhibited the tumor sphere formation of 4T1 cells. The spheres greater than 100 µm in size were enumerated; a representative image of a tumor sphere is shown. The averages of three independent experiments are shown **(A)**. FH535 treatment led to a decrease in the percentage of $CD44^{+}/CD24^{-}$

cells as a proportion of total cancer cells **(B)**. FH535 treatment-mediated cytotoxicity was evaluated by flow cytometry using PE-labeled Annexin-V **(C)**. Cell migration ability was evaluated using the transwell migration assay. FH535 treatment significantly decreased 4T1 cell migration across the membrane in both the upper compartments of transwells compared to vehicle control **(D)**. Abbreviations: TSFE, tumor sphere-forming efficiency. DAPI staining was performed to label the nuclei within each field. The results are the mean ± SD from three independent experiments. * $P<0.05$, ** $P<0.01$, *** $P<0.001$.

Supplement Figure. 6

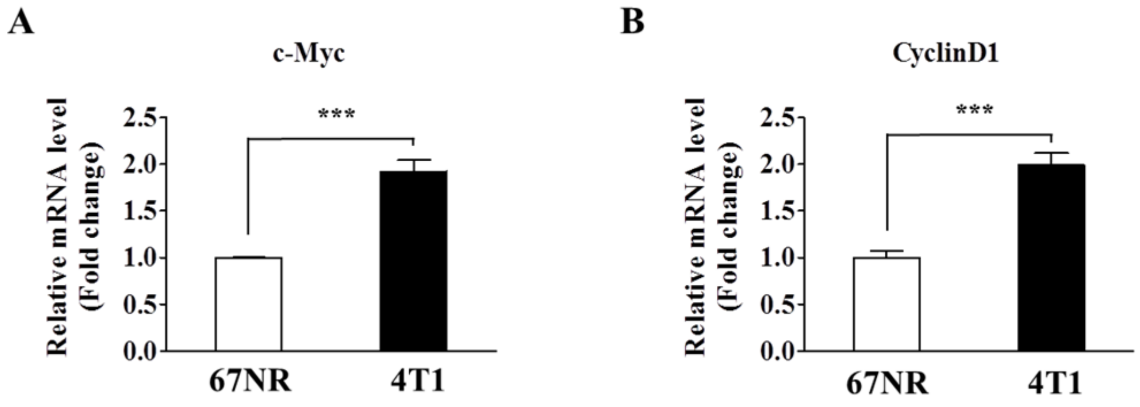

Supplementary figure 6. The significance of Wnt/β-catenin signaling on the metastatic phenotype. The relative expressions of c-Myc and Cyclin D1, which are downstream components of Wnt/β-catenin signaling, in both non-invasive 67NR cells and highly invasive 4T1 cells were evaluated by real-time PCR **(A-B)**. The results are presented as the means ± SD from three independent experiments. * P<0.05, ** P<0.01, *** P<0.001.

Supplement Figure. 7

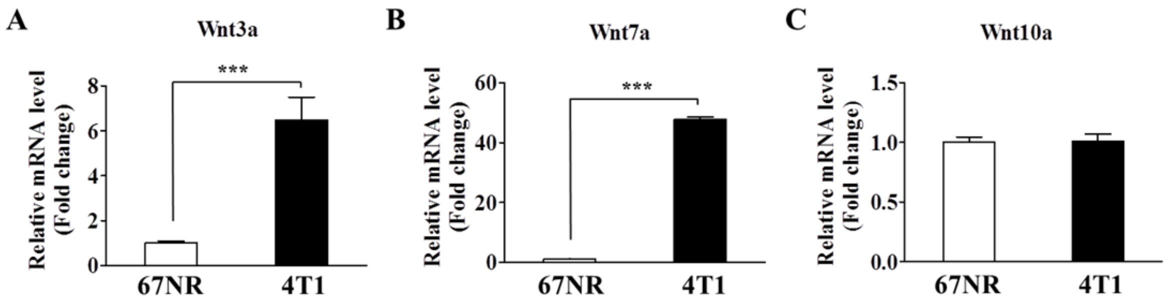

Supplementary figure 7. The expression patterns of Wnt family depend on the metastatic phenotype. The relative expressions of Wnt3a, Wnt7a, and Wnt10a in both non-invasive 67NR cells and highly invasive 4T1 cells were evaluated by real-time PCR **(A-C)**. The results are presented as the means ± SD from three independent experiments. * $P<0.05$, ** $P<0.01$, *** $P<0.001$.

Supplement Figure. 8

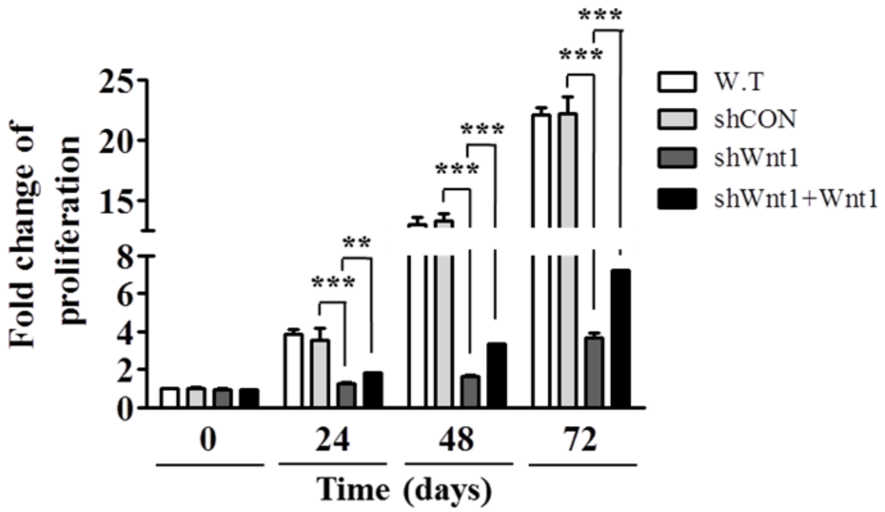

Supplementary figure 8. The specificity of Wnt1 in breast cancer proliferation. 4T1 cells co-treated with Wnt1 ligand with or without Wnt1 knockdown than proliferation was evaluated by MTT assay. Co-treatment of cells with Wnt1 ligand successfully attenuated Wnt1 knockdown-mediated inhibitory effects on the proliferation. The results are the mean ± SD from three independent experiments. * P<0.05, ** P<0.01, *** P<0.001.

Supplement Figure. 9

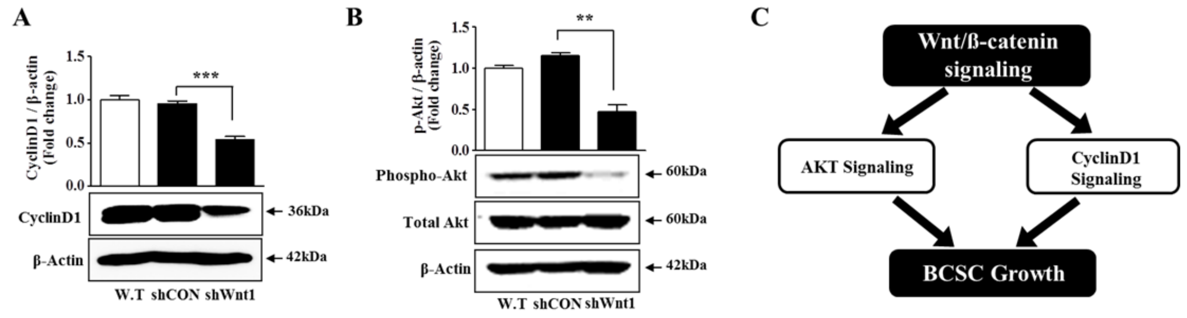

Supplementary figure 9. The effects of Wnt1knockdown on the growth and cycle regulators. The inhibitory effects of Wnt1knockdown on the expressions of Akt and Cyclin D1 were assessed by western blot analysis. β-actin was used as an internal control (**A-B**). The results are presented as the means ± SD from three independent experiments. * P<0.05, ** P<0.01, *** P<0.001.

Supplement Figure. 10

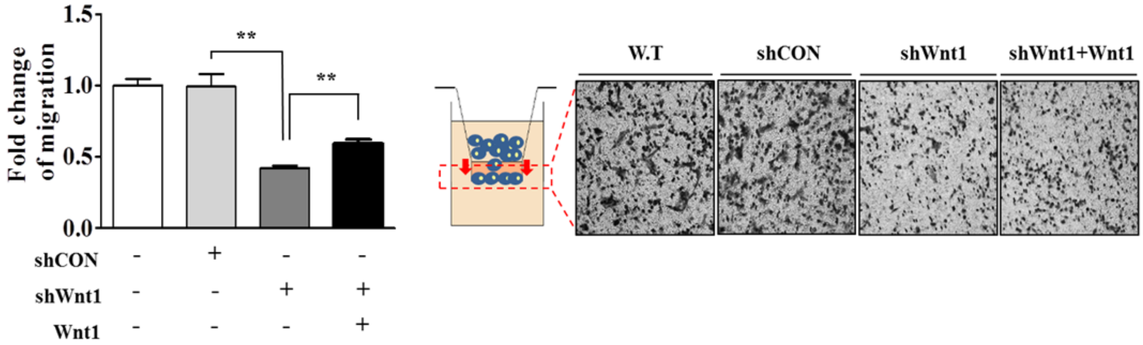

Supplementary figure 10. The specificity of Wnt1 in breast cancer metastasis. Cell migration ability was evaluated using the transwell migration assay. Co-treatment of cells with Wnt1 ligand successfully attenuated Wnt1 knockdown-mediated inhibitory effects on the migration across the membrane in both the upper compartments of transwells compared to control groups. The results are the mean ± SD from three independent experiments. * P<0.05, ** P<0.01, *** P<0.001.

Supplement Figure. 11

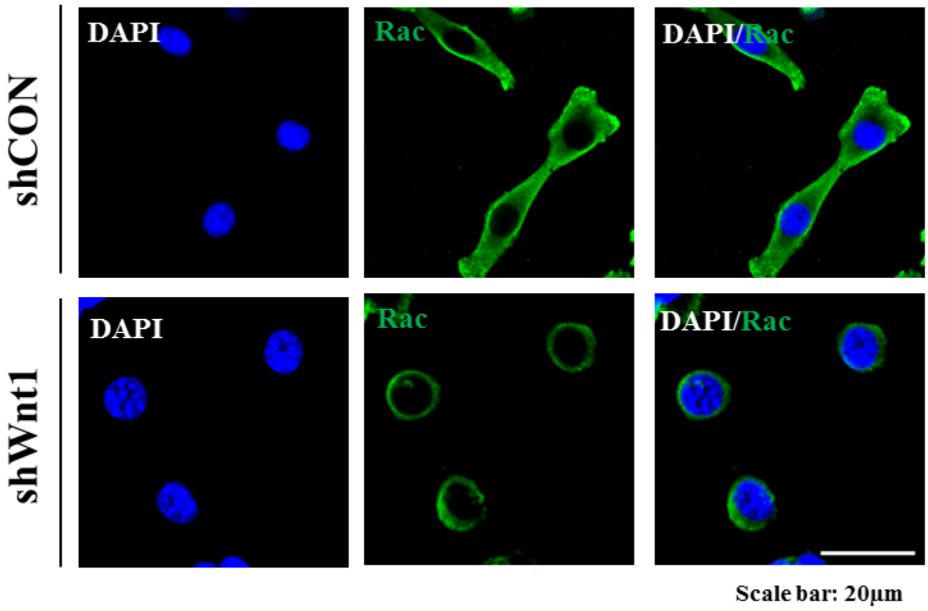

Supplementary figure 11. The effects of Wnt1knockdown on the cytoskeleton disorganization. Wnt1 knockdown-induced fiber disorganization and a full morphological transition were visualized by actin-Rac staining. DAPI staining was performed to label the nuclei within each field.

Supplement Figure. 12

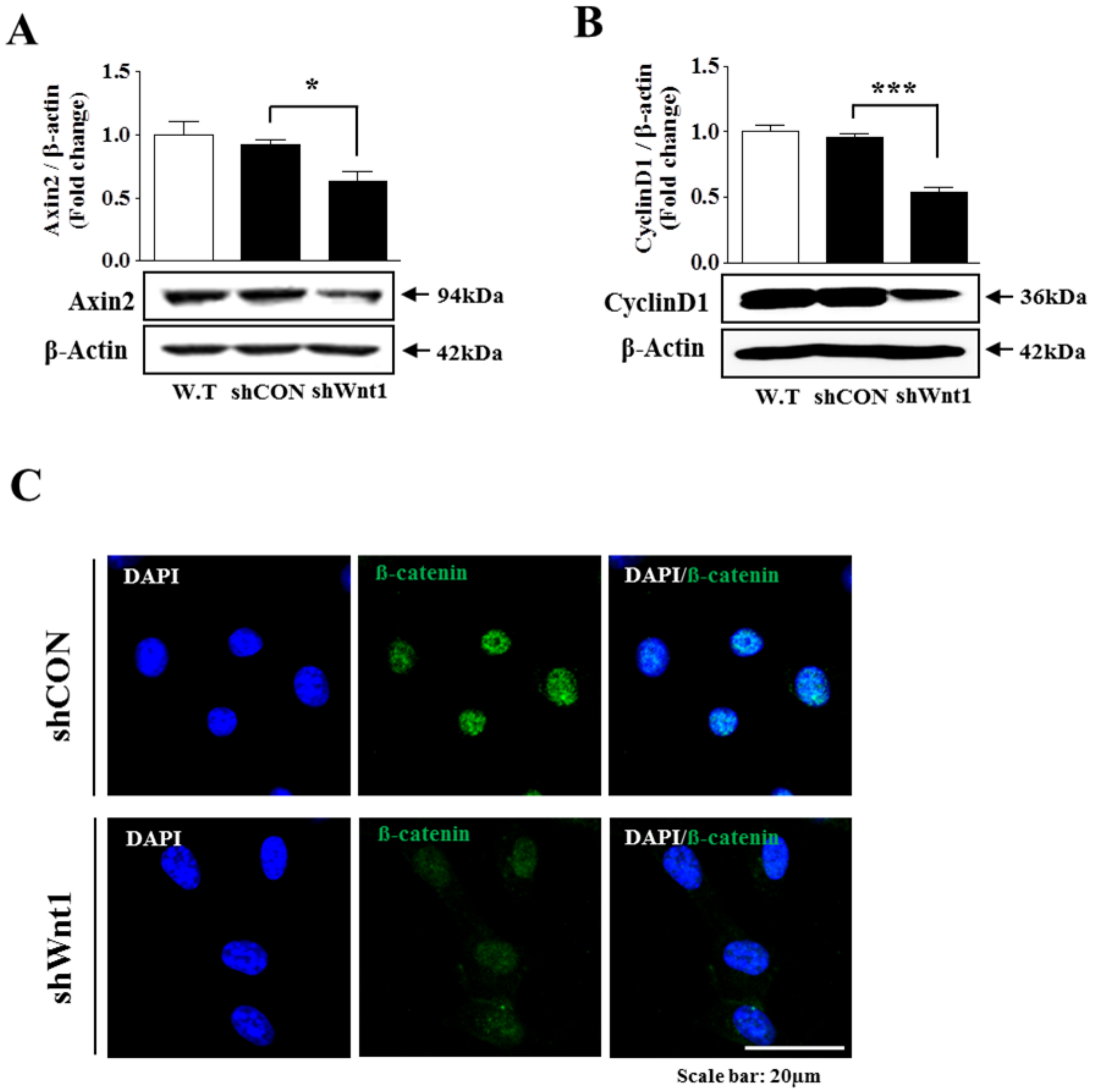

Supplementary figure 12. The effects of Wnt1 knockdown on the β-catenin target genes. The inhibitory effects of Wnt1knockdown on the expressions of Axin2 and cyclin D1were assessed by western blot analysis **(A-B)**. Breast cancer cells were stained using an antibody specific for β-catenin. The nuclear localization of β-catenin is dependent on the Wnt/β-catenin signaling **(C)**. β-actin was used as an internal control. DAPI staining was used to label the nuclei. The results are presented as the means ± SD from three independent experiments. * P<0.05, ** P<0.01, *** P<0.001.

Supplement Figure. 13

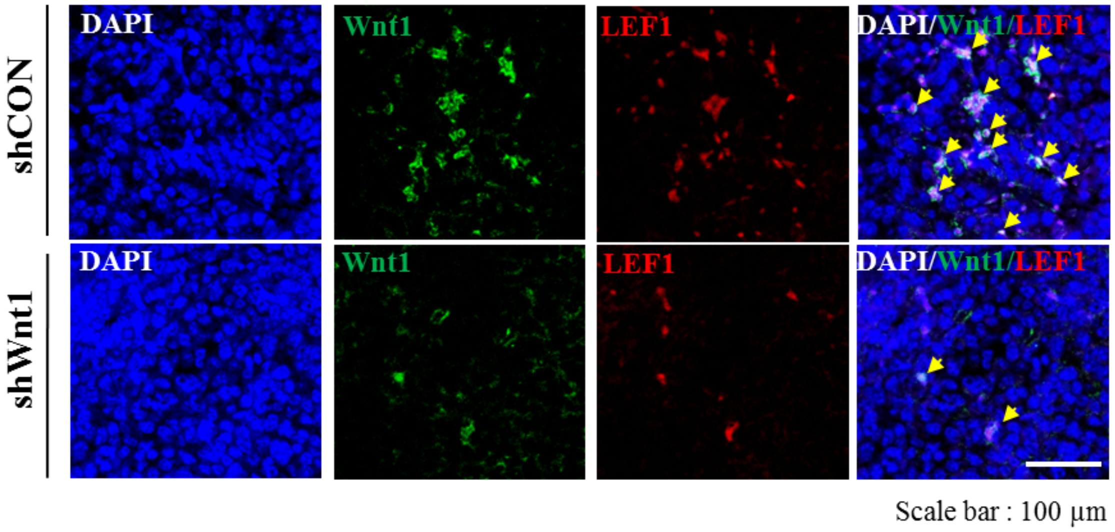

Supplementary figure 13. The co-localization of Wnt/β-catenin signaling components in tumor xenografts. Tumor xenografts were co-stained with antibodies specific for Wnt1 and LEF1.Wnt1-positive populations mostly overlapped with LEF1-positive subpopulations in tumor xenografts. DAPI staining was performed to label the nuclei within each field.

Supplement Table 1. Primer sequences for quantitative RT-PCR

Gene	Genebank No.		Primer sequence
Wnt1	NM_021279	F	GAACCCTTTTGCCATCCTGA
		R	CACCTTCAAGAGTTGACCTC
ß-catenin	NM_007614	F	TGGACCCTATGATGGAGCATG
		R	GGTCAGTATCAAACCAGGCCAG
LEF1	NM_010703	F	TGATTCCTGGTCCCCCTGGC
		R	CACTGTCCGTGTGGGGGTGC
c-Myc	NM_010849	F	CGCACACACAACGTCTTGGA
		R	AGGATGTAGGCGGTGGCTTT
CyclinD1	NM_011806	F	TGCCATCCATGCGGAAA
		R	AGCGGGAAGAACTCCTCTTC
Wnt3a	NM_009522	F	AGCACTCAGCCCATTCTCTG
		R	TCCACGTAGTTCCTGCAGAA
Wnt7a	NM_009527	F	CCGTTGGAACTGCTCAGCG
		R	CCGCAGCGATAATCGCAT
Wnt10a	NM_009518	F	TGGGTAAACTGAAGGCTTGC
		R	CACGGTTGTTGTGGAGTCTC
HPRT	NM_013556	F	GCCTAAGATGAGCGCAAGTTG
		R	TACTAGGCAGATGGCCACAGG
